# Tumor Mutation Burden Correlates With Efficacy of Chemotherapy/Targeted Therapy in Advanced Non–Small Cell Lung Cancer

**DOI:** 10.3389/fonc.2020.00480

**Published:** 2020-04-29

**Authors:** Chen Lin, Xun Shi, Jun Zhao, Qiong He, Yun Fan, Weizhen Xu, Yang Shao, Xinmin Yu, Ying Jin

**Affiliations:** ^1^Institute of Cancer and Basic Medicine (ICBM), Chinese Academy of Sciences, Hangzhou, China; ^2^Department of Medical Oncology, Cancer Hospital of the University of Chinese Academy of Sciences, Hangzhou, China; ^3^Department of Medical Oncology, Zhejiang Cancer Hospital, Hangzhou, China; ^4^Clinical Trials Center, Zhejiang Cancer Hospital, Hangzhou, China; ^5^Nanjing Geneseeq Technology Inc., Nangjing, China; ^6^Zhejiang Key Laboratory of Diagnosis and Treatment Technology of Thoracic Oncology, Hangzhou, China; ^7^Zhejiang Key Laboratory of Radiation Oncology, Hangzhou, China

**Keywords:** tumor mutation burden (TMB), clinical benefit, epidermal growth factor receptor tyrosine kinase inhibitors (EGFR-TKIs), chemotherapy, non–small cell lung cancer (NSCLC)

## Abstract

**Objectives:** Accumulating evidence has illustrated greater benefit of immunotherapy in tumors with high tumor mutation burden (TMB), whereas its impact on targeted therapy or chemotherapy is undefined. Herein, we evaluated TMB outside of immuno-oncology in epidermal growth factor receptor (EGFR)–mutant patients and EGFR/ALK wild-type cohorts.

**Methods:** In this retrospective study, we correlated TMB with response rate and progression-free survival (PFS) of patients who received EGFR–tyrosine kinase inhibitors (TKIs) or pemetrexed/platinum as first-line therapy. Tumor mutation burden was evaluated by targeted next-generation sequencing. Patients were divided into low (L)/intermediate (I)/high (H) TMB groups by tertiles.

**Results:** In EGFR-mutant cohort, TMB-L patients had a massively improved PFS compared to TMB-I and TMB-H patients (16.4 vs. 9.0 vs. 7.4 months; log-rank *p* = 0.006) when treated with first-generation EGFR-TKIs. In EGFR/ALK wild-type cohorts who received pemetrexed/platinum regimen, the objective response rate (ORR) of TMB-L group was statistically superior than that of TMB-I and TMB-H groups (53.8% vs. 23% vs. 8.3%; log-rank *p* = 0.037), and patients with low TMB had a numerically but not significantly prolonged PFS (6.9 vs. 4.3 vs. 4.6 m; log-rank *p* = 0.22).

**Conclusion:** Our data provide insights into the relevance between TMB and targeted/chemo therapy. Higher non-synonymous TMB correlates with inferior PFS for first-generation EGFR-TKIs in EGFR-driven patients and worse response to pemetrexed/platinum regimen in EGFR/ALK wild-type patients, which has potential clinical implications for cancer treatment but needs corroboration in larger studies.

## Introduction

Reliable biomarkers predicting sensitivity or resistance to anticancer therapy aid patient selection for proper therapeutic strategies. For the past decade, non–small cell lung cancer (NSCLC) exemplifies precision medicine with multiple oncogenic molecular alterations that serve as potential targets for therapy, such as epidermal growth factor receptor (EGFR) and ALK, yielding profound clinical benefits to certain cohorts of patients (1, 2). More recently, immunotherapy has transformed the treatment landscape of driver mutation-negative population for the potential of durable responses, which, however, occur in only a minority of patients (3, 4), suggesting the urge for adequate biomarkers of both response and resistance. Figuring out the molecular determinants that predict responsiveness to current antineoplastic agents will largely optimize clinical treatment options.

Tumor mutation burden (TMB), defined as the total number of non-synonymous mutations in the coding regions of genes, is emerging as a promising predictive biomarker for immunotherapy (5, 6). Higher non-synonymous tumor mutation burden has been hypothesized to produce more neoantigens and enhance immunogenicity, which could effectively elicit T cell-mediated anti-tumor response (7, 8). For most cancer histologies, a higher TMB is pertinent to an improved survival in patients receiving immune checkpoint inhibitors (ICIs), although the definition of high TMB, or the cutpoints, varies markedly between diverse tumors (9). To date, the overwhelming majority of research regarding TMB and NSCLC revolves around its predictive capability in immuno-oncology, whereas the role of TMB in other therapeutic strategies is rarely mentioned.

Traditional TMB detection is performed by whole-exome sequencing (WES) as the gold standard in a research setting. However, in terms of cost, time, and availability of formalin-fixed paraffin-embedded (FFPE), it is more inclined to use next-generation sequencing (NGS) diagnostic platforms instead of WES for TMB detection in clinical practice. Tumor mutation burden assessed by large panels was significantly associated with improved benefit among NSCLC patients treated with ICIs and correlated well with WES evaluation (10). Nowadays, the mainstream molecular testing platforms have a wide degree of variance in the NGS gene panels, including Msk-IMPACT (468 cancer-related genes), F1CDx (324 cancer-related genes), Guardant360 (73 cancer-related genes), PlasmaSELECT 64 (64 cancer-related genes), and FoundationACT (62 cancer-related genes) (11). In this retrospective study, TMB was assessed by a 425-gene sequencing panel with matched normal germline sequencing, which has been demonstrated to be strongly correlated with WES and could provide a reasonable estimation of exonic mutation burden (12, 13). We compared TMB level in different subgroups of NSCLC and investigated the impact of TMB on the efficacy of EGFR–tyrosine kinase inhibitor (TKI) or pemetrexed/platinum in advanced NSCLC patients.

## Methods

### Patients

From August 2017 to December 2018, NGS was performed in 198 treatment-naive patients who were diagnosed with advanced NSCLC at Zhejiang Cancer Hospital. Patients' clinical and treatment information was extracted from electronic medical records. The histological classification was based on the World Health Organization criteria (2015 version) (14). Lung cancer staging was performed according to the eighth TNM classification scheme. Study protocols were approved by the Ethical Review Community of Zhejiang Cancer Hospital. All patients provided written informed consent before study entry.

### Sequence Analysis and TMB Calculation

NGS Geneseeq Technology (Nanjing, China) was responsible for the whole NGS as a centralized clinical testing center. Prior to paraffin embedding, all samples were fixed with neutral buffered formalin. Tissue blocks with adequate tumor cellularity (>70%, without significant necrosis or inflammation) were selected by pathologists who were blinded to patients' demographics and clinical data. Briefly, genomic DNA was extracted from 10 to 15 unstained slides of 5 μm-thick tumor FFPE tissues using QIAamp DNA FFPE Kit (QIAGEN, Hilden, Germany). The KAPA Hyper Prep Kit (Kapa Biosystems, Wilmington, MA, USA) was utilized for DNA library preparation as a versatile reagent kit adapted to the Illumina platform (Illumina Inc., San Diego, USA). For hybridization enrichment, customized xGen lockdown probes (Integrated DNA Technologies, Coralville, LA, USA) were applied. The probe panel were designed to target 425 cancer-specific genes. Hybrid Capture Selection was carried out using NimbleGen SeqCap EZ Hybridization & Wash Kit (Roche NimbleGen, Madison, WI, USA) and Dynabeads M-270 Streptavidin (Life Technologies, Darmstadt, Germany). Enriched libraries were amplified and subjected for NGS on Illumina Hiseq4000 NGS platforms. All procedures were conducted according to the manufacturers' protocols.

The NGS panel (Geneseeq) has been demonstrated to be capable of accurately estimating TMB compared with WES with an average size of 1.4 Mb. All base substitutions and indels in the coding region of targeted genes were considered with the exception of known hotspot mutations in oncogenic drivers and truncations in tumor suppressors. Specific somatic mutation calling process was described in previous reports (13). Tumor mutation burden level was stratified by tertiles of low, intermediate, and high according to previous literature (15–18).

### Annotation for Mutations of DNA Damage Response Genes

Thirty-eight genes within 425 cancer-associated genes were identified as DNA damage response (DDR)–related based on published literature and databases such as ClinVar, Catalog of Somatic Mutations in Cancer, and PubMed. DNA damage response–positive cases were defined as any non-silent mutations in gene coding regions, including missense, nonsense, frameshift, start/stop codon changes, and splice site mutations. The detailed profiles of 38 genes involved in eight DDR pathways are listed in Table 1.

**Table 1:** DDR gene panel.

**BER**	**CPF**	**FA**	**HRR**	**MMR**	**NER**	**NHEJ**	**TLS**
MUTYH	ATM	BLM	BRCA1	MLH1	ERCC1	MRE11A	POLH
POLD1	ATR	BRIP1	BRCA2	MLH3	ERCC2	PRKDC	
POLE	CHEK1	FANCA	NBN	MSH2	ERCC3		
PARP1	CHEK2	FANCC	RAD50	MSH6	ERCC4		
PARP2		PALB2	RAD51	PMS1			
		RAD51C	RAD51B	PMS2			
			RAD51D				
			RAD54L				
			RECQL4				
			WRN				

*BER, base excision repair; CPF, check point factor; FA, Fanconi anemia; HRR, homologous recombination repair; MMR, mismatch repair; NER, nucleotide excision repair; NHEJ, nonhomologous end-joining; TLS, DNA translesion synthesis*.

### Assessment of Efficacy

All patients were radiologically evaluable according to Response Evaluation Criteria in Solid Tumors version 1.1 through spiral computed tomography or magnetic resonance imaging scans (19). Progression-free survival (PFS) was defined as the time from the first medication to the first objective progression of disease or the date of death from any causes. Outpatient or telephonic follow-up was adopted, and the last follow-up time of this study was on July 1, 2019.

### Statistical Methods

Statistical analysis was done by GraphPad Prism 6 (version 6, GraphPad Software SanDiego, CA, USA) and SPSS 22.0 (SPSS, Chicago, IL, USA). Comparisons between clinical features and curative effects were tested by χ^2^ test or Fisher exact test. Progression-free survival was estimated by Kaplan–Meier curve and the differences were tested by log-rank test with the hazard ratios calculated using Mantel–Haenszel method. *p* < 0.05 was considered statistically significant.

## Results

### Patient Overview

The demographics and clinical data of 198 NSCLC patients who underwent targeted NGS are summarized in Table 2, including age, tumor stage, pathological type, gender, smoking status, genotype, and TMB. The predominant majority of patients were stage IV (98%) at diagnosis with a median age of 61 years (range, 32–86 years). Lung adenocarcinoma accounted for the main part (168/198, 84.9%), whereas eight patients were squamous cell carcinoma (8/198, 4%), and 22 patients were diagnosed with other pathological types (22/198, 11.1%), including NSCLC not otherwise specified (*n* = 18) and adenosquamous carcinoma (*n* = 4). More than half of the patients were male (58.6%) and non-smokers (53%). Approximately half the patients were EGFR-mutant (98/198, 49.5%), and those who lacked mutations in known targetable genes (EGFR/ALK/BRAF/HER2/KRAS/NRAS/MET//ROS1/RET), known as pan-negative, occupied 17.7% of the cohort. The mutation profiles of all 198 patients are depicted in Figure 1. The gene with the highest mutation rate was TP53, observed in 68.2% of the patients (135/198), followed by EGFR, RB1, ARID1A, and SETD2. Among other driver genes, ALK fusion was the most frequent mutation type sharing a proportion of 7.6% (15/198), followed by KRAS and ERBB2.

**Table 2:** Clinical characteristics of patients at baseline.

**Characteristic**	**All patients (*****n*** **=** **198)**	
	***n***	**%**
**Age, years**		
Median	61	
Range	32–86	
≤65	135	68.2
>65	63	31.8
**Stage**		
IIIB/IIIC	4	2
IV	194	98
**Pathology type**		
Adenocarcinoma	168	84.9
Squamous carcinoma	8	4
Others	22	11.1
**Gender**		
Male	116	58.6
Female	82	41.4
**Smoking history**		
Never smoker	105	53
Ever smoker	93	47
**Genotype**		
EGFR	98	49.5
ALK	16	8.1
E/A-negative driver genes^*^	49	24.7
Pan-negative^#^	35	17.7
**TMB**		
Median	5.6	
Range	0–25.8	

*EGFR, epidermal growth factor receptor; ALK, anaplastic lymphoma kinase; TKI, tyrosine kinase inhibitor; TMB, tumor mutation burden*.

* *E/A-negative driver genes: EGFR/ALK negative driver mutations, including BRAF/HER2/KRAS/NRAS/MET//ROS1/RET*.

# *Pan-negative: tumors that lack mutations in known targetable genes*.

**Figure 1:**
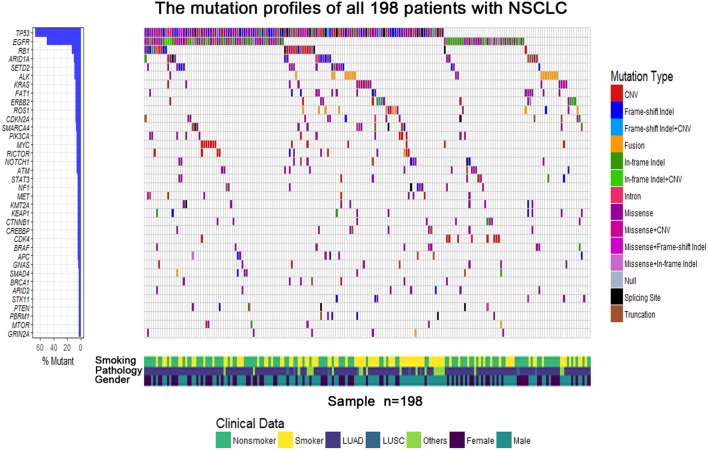
The mutational gene profiles of all 198 patients with advanced non–small cell lung cancer.

### TMB Comparison Between Subgroups

**Figure 2:**
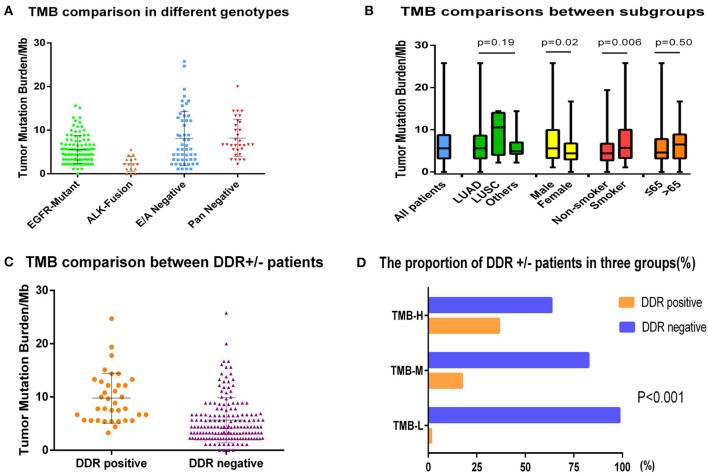
The comparisons of TMB in different subgroups. **(A)** Tumor mutation burden comparison of EGFR-mutant, ALK-fusion, E/A-negative (EGFR/ALK–negative driver mutations, including BRAF/HER2/KRAS/NRAS/MET/ROS1/RET), and pan-negative (tumors that lack mutations in known targetable genes) groups. **(B)** The correlation between demographics or clinicopathologic features and TMB. **(C)** Tumor mutation burden comparison in DDR-positive group and DDR-negative group. **(D)** The proportion of DDR-positive and -negative patients in low (L)/intermediate (I)/high (H) TMB groups.

Tumor mutation burden in the entire cohort ranged from 0 to 25.8 mutations (muts)/Mb with a median value of 5.6 muts/Mb. Because tumors harboring different genes may have distinct biological behaviors, we first divided the population into four subgroups: EGFR-mutant, ALK-fusion, E/A-negative (EGFR/ALK negative driver mutations, including BRAF/HER2/KRAS/NRAS/MET//ROS1/RET), and pan-negative (tumors that lack mutations in known targetable genes). The median TMBs of the four groups were 5.6, 2.3, 8.1, and 8.2 muts/Mb, respectively, displaying a lower TMB in patients with *EGFR* or ALK-fusion mutation compared to wild-type (WT) NSCLC (*p* < 0.001) (Figure 2A). The correlation between other clinicopathologic features and TMB was also analyzed as depicted in Figure 2B. Average TMB in male patients (7.1 muts/Mb) or smokers (7.4 muts/Mb) was statistically higher than in female patients (5.5 muts/Mb) or non-smokers (5.6 muts/Mb) with *p*-values of 0.02 and 0.006, whereas no differences were detected in age and histology subgroups.

We also explored the relationship between DDR genes and TMB. Of 198 patients, 36 (18.2%) were identified as DDR-positive with alterations in the following genes: ATM (33.3%), BRCA1/2 (19.4%), ERCC1/4 (13.9%), RECQL4 (13.9%), ATR (11.1%), WRN (8.3%), BRIP1 (5.6%), NBN (5.6%), MLH1/MSH2/MSH6 (2.8%), and CHEK1/2 (2.8%). Among eight functional categories of DDR pathways, we found that mutations of checkpoint factor were enriched in NSCLC. Furthermore, the median TMB was obviously higher in DDR-positive group compared to the DDR-negative group (9.8 vs. 5.7 muts/Mb, *p* < 0.0001) (Figure 2C). When using the upper-tertile value (>6.7 muts/Mb) to define patients with high TMB, we observed that the DDR mutation occurred more frequently in TMB-H patients than in TMB-I or TMB-L group (23/63 vs. 12/69 vs. 1/66, *p* < 0.001) (Figure 2D).

### TMB and Survival Outcome in EGFR-Mutant Patients

**Figure 3:**
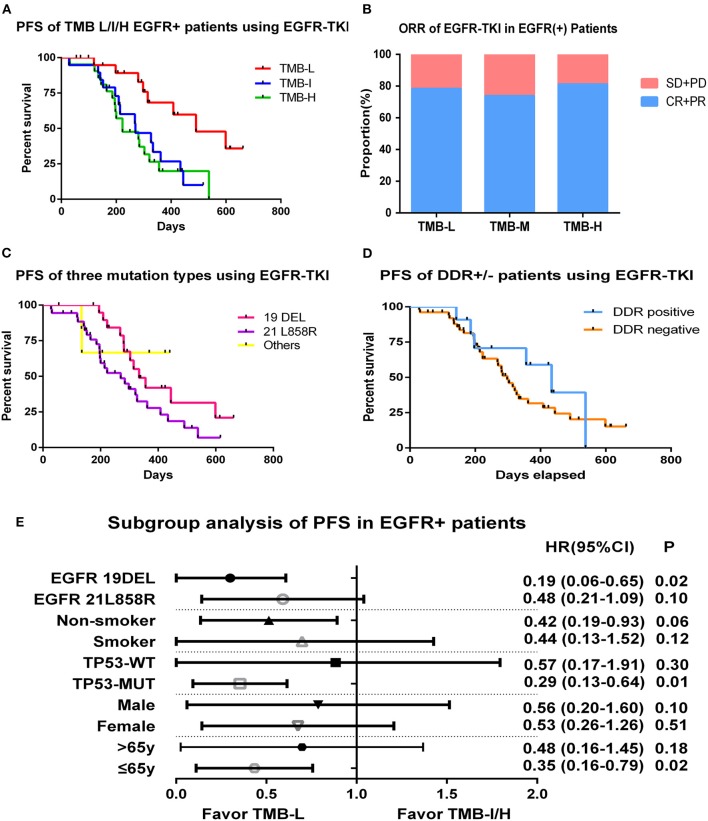
Tumor mutation burden and survival outcome in EGFR-mutant patients. **(A)** Progression-free survival of EGFR-mutant patients using first generation of EGFR-TKIs stratified by tertiles of TMB. **(B)** ORR of EGFR-TKIs in EGFR-mutant patients of TMB L/I/H groups. **(C)** Progression-free survival of EGFR-mutant patients divided by three mutational subtypes. **(D)** Progression-free survival of EGFR-mutant patients divided by DDR genes status. **(E)** Hazard ratio (Mantel–Haenszel method) and *p*-value (log-rank) for subgroups evaluating PFS of EGFR-TKIs in EGFR-mutant patients stratified by low vs. intermediate/high TMB.

Among 98 EGFR-mutant patients, 63 patients who received first-generation EGFR-TKIs (gefitinib or icotinib) as initial treatment and had complete follow-up information were included for survival analysis. Tumor mutation burden in this cohort ranged from 0 to 15.9 muts/Mb with a median value of 5.5 muts/Mb, and the population was divided into three groups by tertiles of TMB: low (≤3.3 muts/Mb), intermediate (3.4–5.7 muts/Mb), and high (>5.7 muts/Mb). We compared the PFS of three groups and found that patients in low TMB group had a superior PFS than in intermediate or high TMB group as 16.4 vs. 9.0 vs. 7.4 m; log-rank *p* = 0.006 (Figure 3A). The objective response rate (ORR) of TMB-L, TMB-I, and TMB-H groups were similar as 78.2, 73.7, and 81.0%, and the disease control rate (DCR)s, were respectively, 100, 94.7, and 95.2% (Figure 3B).

To exclude the influence of other variables on PFS, we performed univariate survival analysis based on gender, age, smoking status, EGFR-mutant subtypes (exon19del vs. L858R vs. others), and TP53 status (WT vs. MUT) and found no factor had an independent effect on PFS except for a trend of superior behavior in EGFR 19DEL cohort (11.1 vs. 9.0m vs. undefined, *p* = 0.09) (Figure 3C). We also explored whether DDR gene alterations were associated with EGFR-TKI sensitivity. Among 63 patients, 11 were DDR positive, and no clear difference was detected between two groups on PFS (Figure 3D). Multivariate analysis were then performed incorporating TMB (low vs. intermediate vs. high), *EGFR*-allele (ex19del vs. L858R vs. others), and *TP53*-status (WT vs. MUT), considering its association with inferior outcomes in previous reports. The results demonstrated low TMB remained significantly correlated with improved PFS compared to the TMB-I and TMB-H groups (*p* = 0.004) (Supplementary Figure 1).

In subgroup analysis, PFS was improved in EGFR 19DEL [hazard ratio (HR), 0.19; 95% confidence interval (CI), 0.06–0.65; log-rank *p* = 0.02], TP53-MUT (HR, 0.29; 95% CI, 0.13–0.64; log-rank *p* = 0.01), and younger patients (HR, 0.35; 95% CI, 0.16–0.79; log-rank *p* = 0.02) in the TMB-L group compared to the TMB-I/H group (intermediate and high TMB groups were combined given their similar outcomes) (Figure 3E).

### TMB and Survival Outcome in Patients Receiving Chemotherapy

**Figure 4:**
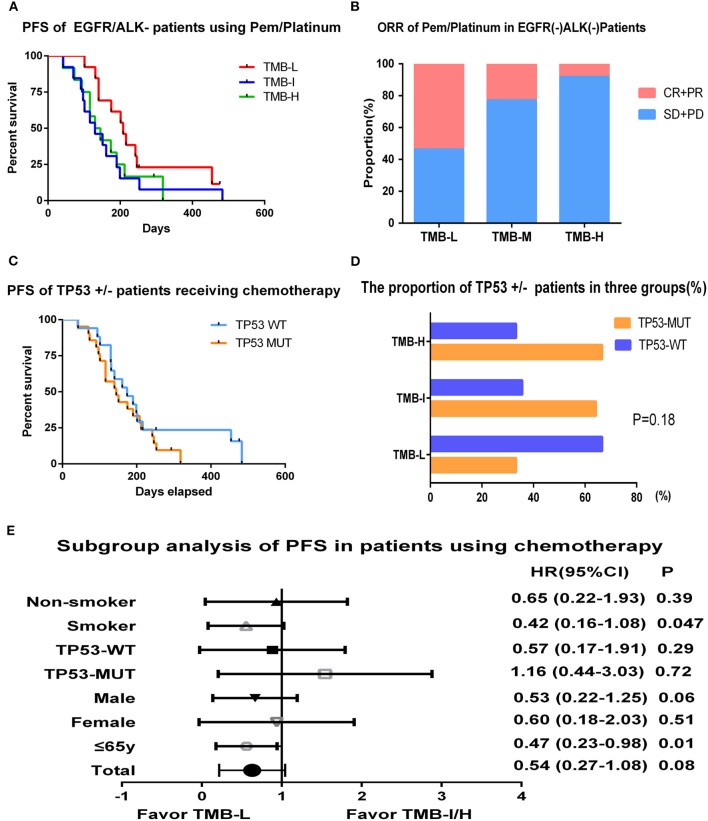
Tumor mutation burden and survival outcome in patients receiving chemotherapy. **(A)** Progression-free survival of EGFR/ALK WT patients using pemetrexed/platinum stratified by tertiles of TMB. **(B)** ORR of pemetrexed/platinum chemotherapy in EGFR/ALK WT patients of TMB L/I/H groups. **(C)** Progression-free survival of EGFR/ALK WT patients divided by TP53 status. **(D)** The proportion of TP53 mutant or WT patients in TMB L/I/H groups. **(E)** Hazard ratio (Mantel–Haenszel method) and *p*-value (log-rank) for subgroups evaluating PFS of pemetrexed/platinum in EGFR/ALK WT patients stratified by low vs. intermediate/high TMB.

Platinum-based chemotherapy containing pemetrexed benefits patients with advanced non-squamous NSCLC substantially. We also explored the correlation between TMB and chemotherapy efficacy in EGFR/ALK WT patients. In our study, a total of 38 lung adenocarcinoma patients were administrated pemetrexed/platinum regimen as first-line therapy. Tumor mutation burden in this cohort ranged from 1.1 to 20.0 muts/Mb with a median value of 6.6 muts/Mb and was also divided by tertiles: low (≤3.2 muts/Mb), intermediate (3.3–7.8 muts/Mb), and high (>7.8 muts/Mb). No obvious differences were detected in demographic characteristics between three groups. The results revealed that patients with low TMB had a numerically but not significantly prolonged PFS than the intermediate or high TMB group (6.9 vs. 4.3 vs. 4.6 m; log-rank *p* = 0.22) (Figure 4A). The ORRs of TMB-L group were statistically superior than that of the TMB-I and TMB-H groups as 53.8, 23, and 8.3% (log-rank *p* = 0.037), and the DCRs were, respectively, 100, 92.3, and 91.7%, respectively (Figure 4B).

TP53 was the most frequent mutation gene in this cohort, occurring in 55% of the patients (21/38). Overall, 17 patients who were TP53-WT had a mild prolonged PFS than TP53-MUT group (5.8 vs. 4.8 m, *p* = 0.37) (Figure 4C). We also examined the distribution of TP53 in the context of TMB and found patients with low TMB had a lower frequency of TP53 mutation (4/12, 33.3%) compared with TMB-I (9/14, 64.3%) and TMB-H (8/12, 66.7%) with a *p*-value of 0.18 (Figure 4D).

In subgroup analysis, PFS was improved in smokers (HR, 0.42; 95% CI, 0.16–1.08; log-rank *p* = 0.047) and younger patients (HR, 0.47; 95% CI, 0.23–0.98; log-rank *p* = 0.01) in the TMB-L group compared to the TMB-I/H group (intermediate and high TMB groups were combined given their similar outcomes) (Figure 4E).

## Discussion

Numerous studies have demonstrated a strong association between TMB and clinical outcomes of cancer patients receiving immunotherapy. Herein, we provided new insights into the role of TMB in targeted therapy and chemotherapy applied in patients with different molecular profilings. Collectively, ALK-fusion patients had the lowest TMB, and pan-negative patients had the highest TMB. DNA damage response–positive patients presented a significantly higher TMB compared to DDR-negative patients. Higher TMB correlates with inferior PFS for first-generation EGFR-TKIs in EGFR-driven patients and worse ORR to pemetrexed/platinum regimen in EGFR/ALK WT patients.

In our study, patients harboring EGFR mutations or ALK fusions had obviously lower TMB than patients harboring no driver genes, which was in concordance with the mainstream opinion that for patients with positive driver genes the TMB was usually lower because there was already a dominant gene in this type of cancer, and in patients with high TMB, the driving genes were mostly negative. This might be partly explained by that oncogenic mutations such as EGFR and ALK were inclined to occur in never-smoker Asian females, whereas never-smoker status was associated with low TMB (20).

The DDR system is essential to preserve genomic integrity and is linked to innate immune signaling to withstand pathogens such as damaged DNAs (21, 22). DNA damage response gene alterations are now attracting increasing attention for the positive correlation with elevated TMB and improved clinical outcomes of programmed death 1 (PD-1)/programmed death 1 ligand (PD-L1) axis inhibition in various cancers (23, 24). Additionally, it has been demonstrated that tumors with deleterious DDR mutations manifested a better responsiveness to platinum-based therapy in bladder cancers (25, 26). In our study, actually, DDR-positive patients had an apparently higher TMB than DDR-negative patients. We also attempted to explore whether DDR status would impact the efficacy of pemetrexed/platinum in NSCLC patients but failed on account of insufficient DDR-positive patients who underwent chemotherapy. Also, it is noteworthy that we here did not distinguish whether the DDR gene mutations were functional on account of insufficient information concerning the functions of various mutations and the lack of hotspots in DDR gene mutations. More elaborate studies with larger cohorts and customized gene panels are required to better illustrate DDR pathways.

At present, the role of TMB in predicting response to EGFR-TKIs or survival was poorly studied except for sporadic reports. Thompson et al. (27) assessed the prognostic significance of ctDNA molecular heterogeneity and found more variants (≥3) detected in plasma were associated with poor overall survival in metastatic lung cancers. Blakely et al. (28) found that clinical non-response to EGFR-TKI was associated with higher alteration burden detected by cfDNA in EGFR-mutant NSCLC. Analogous results using tissue TMB were reported by Offin et al. (29), claiming that EGFR-mutant lung cancer patients with high TMB had shorter time to treatment discontinuation and OS compared to patients with low/intermediate TMB. As a contrast, our result highlighted TMB-L patients had a significantly improved PFS compared to TMB-I/H patients. Although the response of intermediate TMB group differed in two studies, the trend that TMB was negatively associated with clinical outcomes of EGFR-TKIs in advanced EGFR-mutant patients remained consistent. Considering PFS of the first-generation EGFR-TKIs was shorter in patients with high TMB, combination therapy of TKIs with chemotherapy or antiangiogenesis agents, or first-line osimertinib treatment, may be better choices for them (30–32).

The effects of concurrent genetic alterations on clinical outcomes in advanced lung cancers with a primary oncogenic driver have not been fully elucidated. We examined the distribution of common comutation, TP53, in the pre–EGFR-TKI samples in the context of TMB. However, on account of the small sample size, we did not evaluate the role of specific mutations that may correlate with shortened PFS on EGFR-TKIs, such as PIK3CA and MET. Wang et al. (33) reported the coexistence of PTEN loss and increased MET copy number may confer the primary resistance to EGFR-TKIs. Blakely et al. (28) demonstrated co-occurring alterations in CTNNB1 and PIK3CA would cooperatively promote tumor metastasis or limit EGFR-TKIs response. New pathways including WNT/b-catenin alterations and cell-cycle-gene (CDK4 and CDK6) mutations would also diminish EGFR-TKI response (28). Because NGS enables us a full view of genetic landscape in NSCLC, taking other molecular alterations into account, if possible, when analyzing the correlation of TMB and clinical benefits of EGFR-TKIs may be more rational.

Currently, the overwhelming majority of EGFR/ALK WT Chinese patients would descend into chemotherapy in clinical practice considering the huge costs of immunotherapy, whereas the relationship between TMB status and response to chemotherapy is largely unknown. Devarakonda et al. (17) reported that lung cancer–specific survival benefit of adjuvant chemotherapy was more pronounced in patients with low non-synonymous TMBs. Pai et al. (34) revealed that low TMB might be a predictive biomarker in a subset of CRC patients treated with chemotherapy, and irinotecan-based chemotherapy seemed to bring more benefits to patients with low TMB compared to oxaliplatin. Herein, TMB H/I patients who have received pemetrexed/platinum regimen had lower ORR and decreased PFS, although not statistically significant, potentially due to limited population. For these patients, the addition of antiangiogenic therapies to platinum-based chemotherapy, like bevacizumab, or application of anti–PD-1/PD-L1 therapy may be appropriate to bring more survival benefits (35, 36). To the best of our knowledge, this is the first report on the TMB and outcomes with chemotherapy in EGFR/ALK WT NSCLC in first-line metastatic setting. Whether low or high TMB patients respond dissimilarly to other chemotherapy agents of NSCLC deserves in-depth study.

The optimal cutoff value of TMB remains controversial. In this study, TMB was divided by tertiles with reference to a series of classic literature evaluating the value of TMB in immuno-oncology. Some studies have defined the cut-off as the median TMB value (37, 38). Using this approach, we still observed a significant correlation between low TMB and PFS (12.1 vs. 9.0 m; log-rank *p* = 0.018) in EGFR-mutant patients with a median TMB of 4.6 muts/Mb (Supplementary Figure 2). In patients receiving chemotherapy, low TMB group also had a superior ORR as 42% vs. 16% (log-rank *p* = 0.07) and a numerically prolonged PFS than the high TMB group (6.6 vs. 4.3 m; log-rank *p* = 0.07) (Supplementary Figure 3). The conclusion was mainly consistent with previous results, indicating the cutoff selection of TMB as median or tertiles may have little influence on our population.

With the going deep of the research work of TMB, more recent data have complicated its use as an immunobiomarker (39, 40). In Merck's two clinical trials, KEYNOTE 189 and KEYNOTE 021, TMB levels were not significantly correlated with ORR, PFS, and OS. In addition, the complete findings from part 1 of CheckMate 227 also revealed a similar OS benefit for nivolumab and ipilimumab regardless of TMB (stratified at 10 muts/Mb) (41). After these data were released, whether TMB could predict immune efficacy has become confusing. Actually, TMB alone does not represent a direct evidence of tumor immunogenicity, and the optimal assay and cutoff for TMB are not clear. However, this does not disavow the significance of TMB but urges us to be more cautious in the exploration of biomarkers and to have a comprehensive recognition of it. Overall, our study sheds new light on the role of TMB beyond immuno-oncology, yet other exploration space exists, like the assessment of blood TMB. In cases where clinical tissues are difficult to obtain, ctDNA samples from peripheral blood can be used for testing. Blood tumor mutation load test has become a new research hotspot due to its non-invasiveness, convenience, tissue specimen availability, and dynamic monitoring, and it has recently been demonstrated to be feasible to correlate with TMB estimated from tumor tissues (11, 42). The other is integrated multiomic prediction of immunoresponse. For the moment, variables that predict the response to ICIs are mainly categorized into three distinct classes: tumor neoantigens, tumor microenvironment and inflammation, and the checkpoint targets. Integrating certain genomic or pathologic biomarkers may optimize prediction value. For example, Lee and Ruppin (43) constructed a 3-key variable (TMB, estimated CD8^+^T-cell abundance, and the fraction of samples with high PD-1gene expression) model to predict anti–PD-1/PD-L1 response across different cancer types. Cristescu et al. (44) revealed that TMB and inflammatory biomarkers (T cell–inflamed gene expression profile and PD-L1 expression) could jointly predict clinical response to pembrolizumab in a wide range of tumor types. Hence, going beyond a single variate model may be logical to guide selection of suitable patients.

Several limitations of this retrospective study should be acknowledged. First, non-synonymous TMB inferred from a limited gene panel in our study could not achieve accurate assessment such as whole-exome or whole-genome sequencing data. Second, the comparatively small sample size in chemotherapy group might limit the statistical power of this analysis and preclude us from drawing comparisons in certain cohorts. Additionally, the population was only of Asian origin, and the first-line therapy in China for EGFR-mutated lung cancer differed from the United States and other places, which limit the applicability and generalization of the data. In summary, our data provide insights into the relevance between TMB and targeted therapy/chemotherapy. Larger studies are warranted to confirm these results and to delve into the mechanisms underlying this association.

## Data Availability Statement

All datasets generated for this study are included in the article/Supplementary Material.

## Author Contributions

Research design: XY, YF, and YJ. Data collection: JZ and QH. Data analysis and writing: CL, XS, and WX. NGS technical support: YS.

## Conflict of Interest

YS was employed by the company Nanjing Geneseeq Technology Inc., Nangjing, China. The remaining authors declare that the research was conducted in the absence of any commercial or financial relationships that could be construed as a potential conflict of interest.

## Supplementary Material

Supplementary Figure 1 Multivariate analysis incorporating TMB (low vs. intermediate vs. high), *EGFR*-allele (ex19del vs. L858R vs. others), and *TP53*-status (WT vs. MUT).

Supplementary Figure 2 PFS of EGFR-mutant patients using first generation of EGFR-TKIs stratified by median TMB.

Supplementary Figure 3 PFS of EGFR/ALK wild-type patients using Pemetrexed/Platinum stratified by median TMB.
