# Supplementary figures and images for: Tumor Mutation Burden Correlates With Efficacy of Chemotherapy/Targeted Therapy in Advanced Non–Small Cell Lung Cancer
Source: Front Oncol. 2020 Apr 29;10:480. doi: 10.3389/fonc.2020.00480 (PMC7201001; doi:10.3389/fonc.2020.00480)

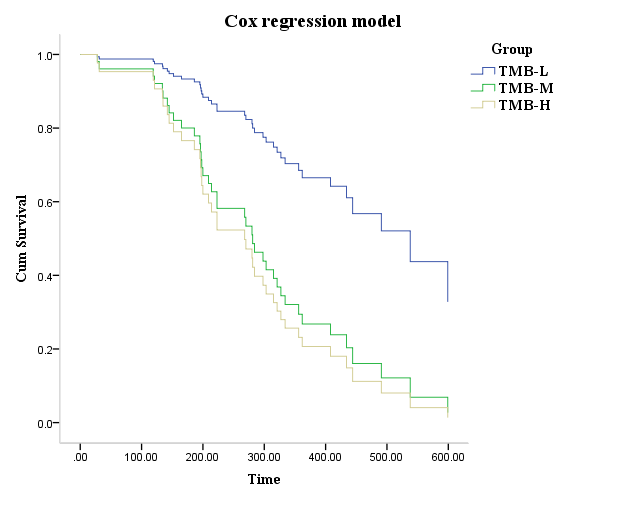

Supplement: Supplementary Figure 1 — Multivariate analysis incorporating TMB (low vs. intermediate vs. high), EGFR-allele (ex19del vs. L858R vs. others), and TP53-status (WT vs. MUT). [file Image_1.PNG]
